# Effectiveness and Student Perceptions of Haptic Virtual Reality Simulation Training as an Instructional Tool in Pre-Clinical Paediatric Dentistry: A Pilot Pedagogical Study

**DOI:** 10.3390/ijerph20054226

**Published:** 2023-02-27

**Authors:** Nebu Philip, Kamran Ali, Monty Duggal, Hanin Daas, Hani Nazzal

**Affiliations:** 1 College of Dental Medicine, QU Health, Qatar University, Doha P.O. Box 2713, Qatar; 2 Hamad Dental Center, Hamad Medical Corporation, Doha P.O. Box 3050, Qatar

**Keywords:** dentistry, education, haptic technology, paedodontics, virtual reality

## Abstract

Simulation training for invasive dental procedures is a core component of the pre-clinical dental curriculum. Besides conventional mannequin-based simulators, dental schools are now incorporating haptic virtual reality simulation (HVRS) devices to facilitate the transition of students from the simulated dental learning environment to the clinical settings. This study aimed to assess student performance and perceptions of HVRS training as a pedagogical tool in pre-clinical paediatric dentistry. After practicing the primary molar pulpotomy procedure on plastic teeth, participants were randomized into test and control groups. Test group students performed the same procedure on a HVRS device, namely the SIMtoCARE Dente^®^. Subsequently, both the test and control group students attended another conventional pulpotomy simulation session where the quality of their access outline and pulp chamber deroofing steps were evaluated on plastic teeth. After the control group students also experienced the HVRS, all study participants completed a perception questionnaire on their experience. No significant differences were found between the study and control group students for the quantitative parameters assessed. Although the students regarded HVRS to be a useful adjunct to support their pre-clinical training, an overwhelming majority of the students did not consider HVRS to be a replacement for conventional pre-clinical simulation training.

## 1. Introduction

Undergraduate dental education requires students to demonstrate pre-clinical competence in a variety of irreversible operative procedures prior to their clinical placement. Dental education programs are time- and place-bound which adds pressure on educators to assist students to achieve the requisite fine motor skills before they perform invasive clinical procedures on patients. Traditionally, dental schools have relied on pre-clinical simulation training using plastic teeth mounted on mannequins to optimise students’ practical skills and hand-eye-foot co-ordination. However, practicing dental procedures on virgin plastic teeth does not accurately simulate patient cases encountered in clinical practice and poses some limitations on the translational value of pre-clinical training. Moreover, plastic teeth do not replicate enamel and dentine hardness and it is difficult for students to receive realistic tactile feedback. The risk of injury associated with the use of sharp instruments during these sessions mandates close supervision which further restricts and reduces the number of pre-clinical simulated sessions that can be offered to students. With dental students required to show competency in increasingly complex procedures, conventional standalone mannequin simulators do not offer an ideal solution and technological advances are required to address their limitations [1].

Dental schools around the world are now increasingly incorporating virtual reality simulators into pre-clinical training to facilitate the transition of students from the simulated dental learning environment into clinical settings. The experience of using virtual reality simulators has been reported to be more pleasurable and enjoyable by early year dental students [2]. These technologies create a virtual environment that is safe and allows students to repeatedly practice clinical procedures in their own time while receiving synchronous standardized computer-generated feedback. The self-assessment process helps students to identify individual learning needs and to engage in self-directed learning and critical thinking [3]. Despite the high initial cost of virtual reality simulators, the absence of procedure repetition limits, fewer consumables required, and reduced faculty supervision time has been shown to have long-term cost benefits [4,5,6]. Furthermore, real-time, process-based recordings of a performed dental procedure allows students and their instructors to review errors at the exact time point they occurred for detailed feedback and correction [7]. A more recent innovation, combines haptic technology with virtual reality simulation in the form of haptic virtual reality simulators; a cutting-edge technology that has revolutionised dental education globally [8]. Haptic virtual reality simulation (HVRS) offers sensory (haptic) feedback in the form of pressures, vibrations, and sounds, allowing students to feel dental instruments and oral tissues in a virtual environment and perform clinical procedures with realistic force feedback [9].

Several questions remain concerning the role and place HVRS should be given in pre-clinical dental education courses. Studies indicate that HVRS introduced in the early stages of undergraduate dental education has greater potential of predicting subsequent clinical performance scores compared to mannequin-based typodont models and also facilitates psychomotor skill acquisition [4,10]. However, for pre-clinical exercises in restorative dentistry and endodontics, the majority of studies suggest that training on HVRS and conventional mannequins had equivalent effects on test scores, procedural errors, dexterity levels, and student learning curves [1,2,6,11,12,13,14,15,16]. For paediatric dentistry, virtual reality simulation training was found to significantly improve dental students’ skills in expressing empathy, behaviour management, and local anaesthesia delivery [17,18,19,20]. However, there is limited research comparing HVRS and conventional mannequin-based simulation training for pre-clinical paediatric dentistry operative procedures, with data from a single study suggesting that students were more comfortable with the conventional simulation training setup than the HVRS [21].

Pre-clinical training on operative skillsets in simulated dental learning environments is a fundamental educational strategy to ensure dental students demonstrate appropriate clinical procedural skills before they undertake invasive procedures in real patients. Training in simulated settings offers a safe environment to develop and consolidate core skills without the risk of harming patients. Management of paediatric dental patients poses additional challenges due to the anxiety within dental care settings often observed in young patients. Dental students often need additional time to calm children and establish a rapport with them using appropriate behaviour management techniques [21]. Dental students also need the skills to efficiently complete complex paediatric dental procedures in a timely manner due to the limited attention span and/or cooperation of children undergoing dental treatment. Thus, it is imperative for dental educators to enhance the clinical skills and confidence of dental students in performing paediatric restorative procedures by employing the most appropriate pre-clinical training methods.

There is clearly a need for more studies to evaluate the effectiveness and student perceptions of training with the HVRS compared to the conventional mannequin simulator for pre-clinical operative procedures in paediatric dentistry. Therefore, the aim of the study was to assess whether augmenting the conventional simulation environment (CSE) with HVRS is likely to affect student performance and experiences for performing the primary molar pulpotomy procedure. The research objectives of the study include: (i) to evaluate if augmenting the CSE pre-clinical training with HVRS for a primary molar pulpotomy can improve student performance scores and reduce procedural time in comparison to pre-clinical training only in the CSE; and (ii) gauging student perceptions of HVRS training for the pre-clinical primary molar pulpotomy procedure.

The conceptual framework of the study was built on the theory of deliberate practice which emphasizes embedding a self-reflective feedback loop in the development of skills rather than simply performing a task repetitively to achieve mastery [22]. Students are at the heart of activities in the simulated dental learning environment and delivery of education should be informed by students’ prior experiences, learning needs, preferences, and expected competencies [23]. Deliberate practice should focus on the quality of learning rather than simply performing a task repeatedly. Student learning should focus on their abilities to process and integrate information to achieve competence in specific skills. With regards to paediatric dentistry, dental students are expected to integrate their knowledge on tooth anatomy, pathology and operative dentistry and apply it to the management of dental diseases in children. Practical training in operative dental procedures is not limited to demonstrating skills in completing a given procedure but, more importantly, mandates the learners to adopt a safe approach to ensure that they do not cause any damage to the teeth.

## 2. Materials and Methods

### 2.1. Ethics Approval

The study protocol and participant consent forms were approved by the Institutional Review Board of Qatar University (Reference number: QU-IRB 1652-EA/22). Prior to the study, full disclosure of the study and participants rights were given verbally and written informed consent was obtained. Students were clearly informed that participation in the study is optional and would in no way affect their training or assessment.

### 2.2. Settings

Qatar University College of Dental Medicine.

### 2.3. Study Design

Interventional randomised controlled pilot study.

### 2.4. Participants

The entire batch of Year 4 undergraduate dental students at the Qatar University College of Dental Medicine consented to participate in the study. The total number of students who participated in the study was 14, the mean age of the students was 22.4 years, and the female/male proportion was 12:2. Prior to the study, the students had received three credit hours of paediatric dentistry training that included attending didactic lectures and completing pre-clinical preventive and restorative exercises in primary teeth. All the participants had previous experience of using the HVRS and the mannequin simulator for pre-clinical restorative and endodontic procedures in permanent teeth.

### 2.5. Study Protocol

**Figure 1:**
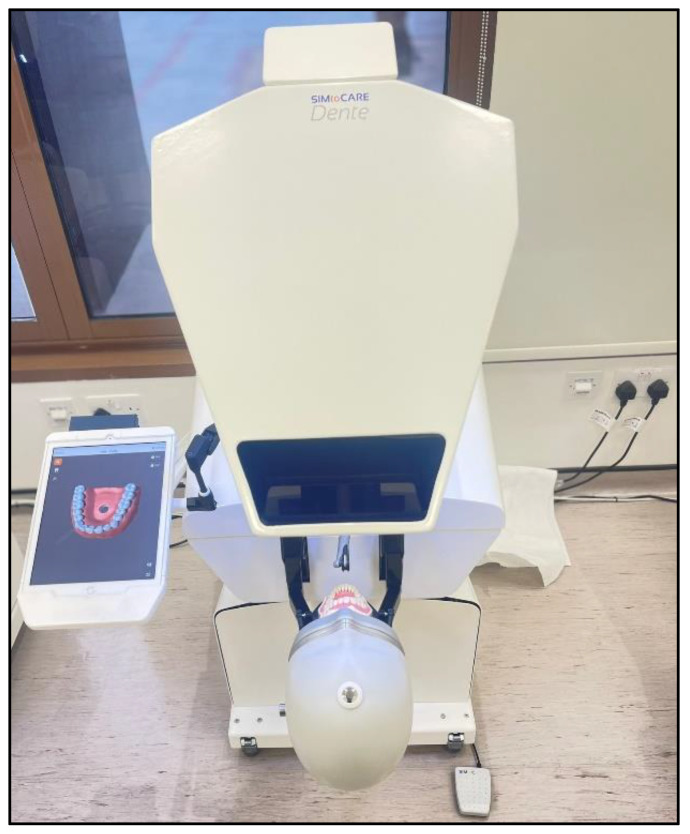
SIMtoCARE Dente^®^ Haptics Reality Virtual Simulator.

The SIMtoCARE Dente^®^ (SIMtoCARE B.V., Vreeland, The Netherlands) was the HVRS device used in the study (Figure 1). SIMtoCARE Dente^®^ provides multimodal feedback particularly touch (haptic) feedback via a physical handpiece with a virtual tip and a dental mirror handle. These physical instruments are graphically modelled on a display monitor along with the realistic images of the teeth and the jaws. Handpiece speed is controlled by a real foot pedal and the handpiece is connected to a force feedback robotic arm giving haptic tactile sensations of real tooth preparations and aerator sound renderings. This system creates a representative and virtual dental environment allowing users to feel tactile sensations experienced when drilling with the dental bur for procedures such as removal of caries or cavity/crown preparations. The SIMtoCARE Dente^®^ is equipped with bespoke ‘Courseware’ software (ACTA, The Academic Centre for Dentistry, Amsterdam, The Netherlands) that includes a ranges of manual dexterity exercises and dental operative procedures of varying complexity. For this study, the pulpotomy exercise on a carious primary molar (tooth #85) was chosen from the Courseware package. 

**Figure 2:**
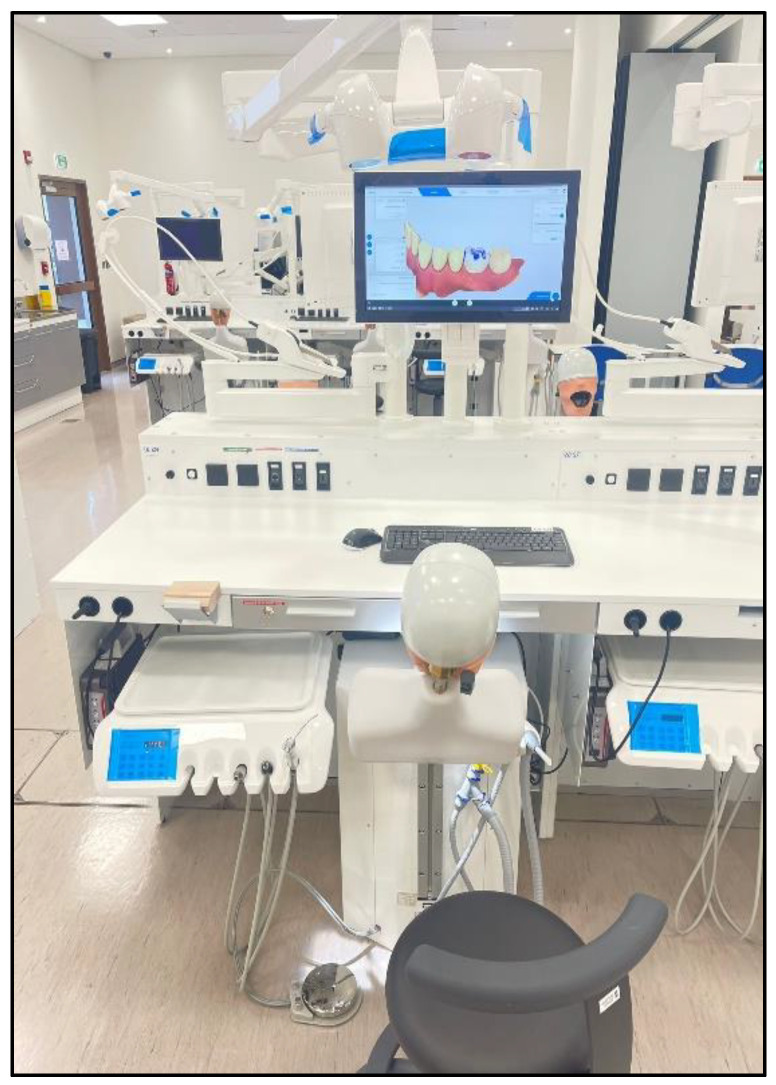
Conventional Mannequin Simulator.

The conventional simulator used in this study was a mannequin head with rubber cheeks that was fitted with replaceable plastic jaws (Frasaco Pedodontal Model AK-6/2 DAK, Tettnang, Germany). This simulator setup uses actual dental instruments including high- and low-speed handpieces and suction devices (Figure 2). For this study, a replaceable plastic primary tooth (tooth #85; Frasaco AK 6/2 ZPUW, Tettnang, Germany) with red wax inserts in the pulp chamber was used for the pulpotomy exercise.

### 2.6. Data Collection

All students participating in the study received a 60-min didactic lecture on pulpotomy in primary teeth. The students also attended two practical demonstrations of how to perform the pulpotomy procedure on a plastic primary tooth mounted on the mannequin simulator and on a virtual carious primary tooth in the SIMtoCARE Dente^®^ simulator. All the students then practiced the pulpotomy procedure on the plastic primary molar over a 90-min session under instructor supervision. The students were then randomly assigned to test (HVRS + CSE) and control groups (CSE) using an online research randomizer tool (www.randomizer.org (accessed on 20 October 2022)). Following this, the test group students practiced the access outline and pulp chamber deroofing steps on the SIMtoCARE Dente^®^ over a single 60-min session. There were no limits placed on the number of attempts the test group students could practice using the pulpotomy procedural steps on the HVRS device during this session. 

The following week, both the test and control group students attended another conventional pulpotomy simulation session and the quality of their access outline and pulp chamber deroofing steps were independently assessed by two examiners who were blinded to the group the students belonged to, based on a defined rubric (Supplementary Table S1). Four-point scores (1: Well-below standard; 2: Just below standard; 3: Meets standard; and 4: Above standard) were used to individually evaluate quality of the access outline and deroofing steps of the pulpotomy procedure. Any disagreement between examiner scores was resolved by re-evaluating and reaching a consensus. For each student, the time taken to complete the two pulpotomy procedural steps on the plastic primary tooth and number of times he/she may have requested instructor help was also recorded. Finally, the control group students were also given an opportunity to experience performing the pulpotomy procedure in the SIMtoCARE Dente^®^ simulator similar to their test group counterparts. 

Once all students completed training in both simulation environments, they were invited to participate in a survey questionnaire recording their experiences and perceptions (Supplementary Table S2). Ten Likert scale questions, comprising five response options (strongly agree; agree; neutral; disagree; and strongly disagree), and four open-ended questions were used to evaluate participants’ experience of the HVRS learning environment. To ensure confidentiality, questionnaires were separated from the consent and information forms and analysed anonymously.

### 2.7. Data Analyses

Normal distribution of data was confirmed using the Shapiro–Wilk test and the equality of variances checked with the two-tailed F-test. Independent sample *t*-tests were used to compare the mean differences in performance scores between the test and control groups. All statistical tests were two-tailed and a *p* < 0.05 was set as the cut-off point to control for alpha error. The statistical analyses were performed using SPSS ver.23 (IBM, New York, NY, USA).

## 3. Results

### 3.1. Student Performance Scores

**Figure 3:**
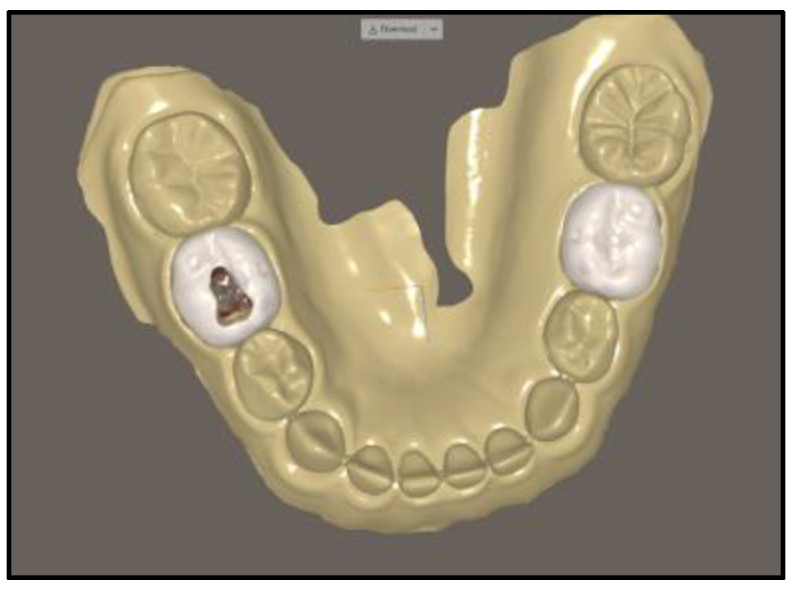
Virtual tooth pulpotomy.

**Figure 4:**
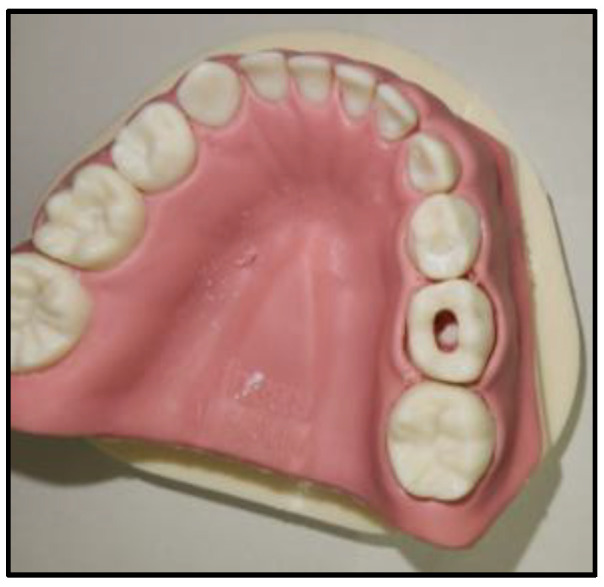
Plastic tooth pulpotomy.

All 14 students in Year 4 participated in the study yielding a 100% response rate. Performance scores for the access outline and pulp chamber deroofing steps did not show any significant differences between the study and control groups. Time taken to complete the procedural steps and number of instructor prompts requested by students also showed no differences between the groups (Table 1). Figure 3 and Figure 4 depict examples of the completed pulpotomy access outline and deroofing procedural steps on the virtual tooth and the conventional plastic tooth, respectively.

### 3.2. Student Perceptions

A questionnaire (Supplementary Table S2) to assess student perceptions of the HVRS was completed by all 14 participants (*n* = 14) once the control group students completed their HVRS session. The student responses to the questionnaire are presented in Table 2. Almost 72% of the students agreed or strongly agreed that images of the teeth, pulp chamber, and instruments displayed on the HVRS monitor looked realistic. However, student responses were more equivocal on whether the tactile sensations and texture/hardness of dental tissues offered by the HVRS device felt realistic. While 43% of the students agreed or strongly agreed that the tactile force feedback provided by the HVRS device felt realistic, 50% of the students suggested that they could not distinguish between enamel and dentine texture/hardness on the SIMtoCARE Dente^®^ simulator. A significant majority of the students (79%) also suggested that deroofing the pulp chamber felt different in the two simulation environments. Approximately, 64% of the students agreed or strongly agreed that training on the HVRS device improved their psychomotor skills and confidence in performing the pulpotomy procedure. Nevertheless, an overwhelming majority of students (86%) disagreed or strongly disagreed with the statement that HVRS training can replace conventional pre-clinical training on typodont teeth for the pulpotomy procedure, with most suggesting that HVRS may be used as an adjunct to the conventional simulation training. Just over half the students (57.1%) expressed interest for more HVRS sessions for paediatric pre-clinical pulp therapy procedures.

Student responses to the open-ended questions suggested that the main benefits of training on the HVRS device was that it allowed more repetitions for practice and provided better visualization of pulp chamber and root orifices. Among the HVRS limitations was the lack of finger rests and the lag in navigating through the displayed images while performing the procedure. Most of the students also preferred to experience the HVRS training after the conventional simulation training. Student recommendations for improving pre-clinical training for the primary molar pulpotomy procedure included providing them an opportunity to practice the procedure on extracted primary teeth and scheduling extra pre-clinical sessions.

## 4. Discussion

Procedure-based dental specialties like paediatric dentistry require students to demonstrate high levels of psychomotor skills before they start treating patients in clinics. Consequently, acquisition of psychomotor skills is fundamental to developing competency in operative procedures during pre-clinical training for undergraduate dental students. While mannequin-based simulation training using typodont teeth has conventionally been the standard pedagogical tool used in the pre-clinical teaching curriculum, dental schools have also been investing in virtual reality simulators to further enhance fine motor skills of their students. However, the impact of these digital methods of pre-clinical training on students’ learning experiences in paediatric dentistry needs further investigation. 

Previous studies from around the world have reported low confidence among undergraduate dental students in performing pulp treatment in children and reiterated the need to improve pre-clinical training [24,25,26,27]. The present study explored whether augmenting the CSE with HVRS training for a primary molar pulpotomy procedure can translate into improved performance scores and lesser procedural time in the conventional simulation environment. The study results showed that there were no significant differences in any of the quantitative parameters assessed (access outline/deroofing scores, number of instructor prompts, and procedural time taken) between students who experienced both the HVRS and CSE, and those who experienced only the CSE. These results are consistent with those from previous studies which also reported no differences in performance of students exposed to the virtual simulation environments for restorative and endodontic pre-clinical procedures [1,2,6,11,12,13,14,15,16]. In contrast, a couple of recent studies did show significant improvements in students’ performance for cavity preparations in permanent teeth after HVRS training [8,28].

A plausible reason why no performance score differences were found in this study could be attributed to the fact that study participants had previously experienced both the simulation environments for other pre-clinical tasks in the earlier years of their training. Additionally, the plastic teeth used in this study were customized for the pulpotomy procedure with wax inserts in the pulp chamber, allowing students to ‘feel’ the pulp in both the simulation environments. In this study, study group students were exposed to approximately 60 min of haptic pulpotomy exercises prior to the combined performance evaluation with the control group students on plastic teeth. Whether a longer time for practice on the HVRS device could have significantly improved student performance scores needs further investigation. 

Qualitative data on student perceptions about pre-clinical HVRS training for the primary molar pulpotomy procedure was also collected in this study. The vast majority of the study participants did not consider HVRS training to be an adequate replacement for conventional simulation training and instead preferred the HVRS be used as an adjunct along with conventional mannequin simulators. This is in agreement with the findings reported by Zafar et al. in a similar pre-clinical paediatric dentistry training study of Australian dental students [21]. Despite the encouraging feedback of the participants in this study with regards to the positive effect of the HVRS on their fine motor skills and confidence in performing the pulpotomy procedure, the majority of the students felt that hardness, texture and tactile sensations offered by the HVRS device were not realistic. These results are in agreement with other studies where less than third of the students felt the hardness, texture, and tactile sensations of the HVRS devices to be realistic [21], with students also finding the experience of drilling in plastic teeth to be more ‘real’ than haptic drilling [1,29]. 

The willingness of a large proportion of the students to engage in more HVRS sessions for pre-clinical paediatric dental procedures is of interest. This apparent incongruity could be explained by the fact that the HVRS allows students’ multiple practice attempts with less time pressures as indicated in the student responses to the open-ended questions of the survey. Furthermore, HVRS training allows the students to receive multi-source feedback, that includes not just instructor- and computer-generated feedback, but also the opportunity and time for self-reflective feedback.

Recreating a clinical experience in a simulated environment is challenging and complex, but it remains the core component of the pre-clinical dental curriculum. This makes it important to explore the benefits of different simulation training environments from the students’ perspective and their relevance to actual clinical practice of different dental procedures. To the best of our knowledge, this is the first study to assess both students’ performance scores and perceptions for a pre-clinical pediatric dental procedure in two simulation environments. The randomisation of students and blinding of assessors in this study improve and reduce selection and detection/performance bias, respectively. Despite recruiting 100% of students attending the paediatric dental pre-clinical training, the low number of participants in this pilot study coupled with the use of a single operative procedure might have affected the results of this study. Another limitation of the study was that the unidimensional Likert scales used to assess student perceptions may not have fully captured the entire gamut of student perceptions. This was partly overcome by including four open-ended questions in the questionnaire to obtain a wider range of student opinions. While the study results should not be over generalized, the study does provide preliminary data for dental faculty considering the use of HVRS as a pedagogical tool for pre-clinical paediatric dentistry training. A multi-center assessment of the effect of HVRS as an adjunct to CSE on a larger cohort of students with different operative procedures such as conventional stainless steel crown preparation and restorative techniques such Class II cavity preparation on primary molars could help further investigate the true benefits of augmenting the conventional simulation with HVRS.

## 5. Conclusions

Despite the current generation of students being more comfortable with digital technologies, the initial findings of this pilot study suggest that HVRS should augment rather than replace conventional pre-clinical paediatric dentistry training. Similar evidence has been reported for other pre-clinical procedures, suggesting that students see HVRS as providing a more diverse learning environment, serving as a bridge between the conventional pre-clinical training and the clinics. Well-designed and adequately powered long-term prospective studies exploring matters of student performance, learning outcomes, and cost-effectiveness are warranted.

## Figures and Tables

**Table 1:** Mean values (±SD) for each evaluation criterion of the study and control groups and results of the *t*-test.

Evaluation Criteria					
	Access Outline Scores	Deroofing Scores	Access Outline Prompts	Deroofing Prompts	Procedural Time (min)
Test Group (CSE + HVRS) (*n* = 7)	2.1 ± 1.1	2.7 ± 0.8	0.4 ± 0.5	0.1 ± 0.4	23.7 ± 1 (7.1%)
Control Group (CSE) (*n* = 7)	2.0 ± 0.8	2.3 ± 0.9	0.4 ± 0.8	0.1 ± 0.4	25.9 ± 8.9
*p*-value	0.67	0.37	1.0	1.0	0.63

**Table 2:** Student perceptions of pre-clinical haptic virtual reality simulation training for the primary molar pulpotomy procedure.

Responses n (%)					
Statement	Strongly Agree	Agree	Neutral	Disagree	Strongly Disagree
Pulpotomy demonstration on the HVRS device allowed me to clearly comprehend the tasks expected from me	2 (14.3%)	5 (35.7%)	4 (28.6%)	1 (7.1%)	2 (14.3%)
Images of the teeth, pulp chamber, and instruments displayed on the HVRS monitor looked realistic	4 (28.6%)	6 (42.9%)	3 (21.4%)	1 (7.1%)	0
I could differentiate between the texture and hardness of enamel and dentine in the HVRS device	4 (28.6%)	2 (14.3%)	1 (7.1%)	3 (21.4%)	4 (28.6%)
Tactile force feedback given by the HVRS device felt realistic	1 (7.1%)	5 (35.7%)	5 (35.7%)	1 (7.1%)	2 (14.3%)
Deroofing the pulp chamber on the HVRS device felt similar to that on plastic teeth mounted on mannequins	0	0	3 (21.4%)	6 (42.9%)	5 (35.7%)
Training on the HVRS device improved my fine motor dental skills	1 (7.1%)	8 (57.1%)	3 (21.4%)	2 (14.3%)	0
Training on the HVRS device improved my confidence in performing the pulpotomy procedure	1 (7.1%)	8 (57.1%)	2 (14.3%)	3 (21.4%)	0
HVRS can replace conventional pre-clinical training on plastic teeth for the pulpotomy procedure	0	1 (7.1%)	1 (7.1%)	6 (42.9%)	6 (42.9%)
HVRS can supplement conventional pre-clinical training on plastic teeth for the pulpotomy procedure	4 (28.6%)	7 (50%)	2 (14.3%)	0	1 (7.1%)
I would like to have more HVRS sessions for paediatric pre-clinical pulp therapy procedures	3 (21.4%)	5 (35.7%)	5 (35.7%)	1 (7.1%)	1 (7.1%)

## Data Availability

The data that support the findings of this study are available from the corresponding author upon reasonable request.

## Supplementary Materials

The following supporting information can be downloaded at: https://www.mdpi.com/article/10.3390/ijerph20054226/s1, Supplementary Table S1: Evaluation rubric for access outline and deroofing steps of the primary molar pulpotomy procedure; Supplementary Table S2: Student perceptions of pre-clinical haptic virtual reality simulation training.
