# Supplementary material for: Effectiveness and Student Perceptions of Haptic Virtual Reality Simulation Training as an Instructional Tool in Pre-Clinical Paediatric Dentistry: A Pilot Pedagogical Study
Source: Int J Environ Res Public Health. 2023 Feb 27;20(5):4226. doi: 10.3390/ijerph20054226 (PMC10001601; doi:10.3390/ijerph20054226)
Supplement: Supplementary file 1 [file ijerph-20-04226-s001.zip › ijerph-2211181-supplementary.pdf]

Supplementary Table S1: Evaluation rubric for access outline and deroofing steps of the primary molar pulpotomy procedure

	Well-below standard (Score 1)	Just below substandard (Score 2)	Meets standard (Score 3)	Above standard (Score 4)
Access outline	• Failure to achieve correct access outline despite being prompted<br>**AND/OR**<br>• Major damage to tooth structure	• Able to achieve correct access outline with prompts<br>**AND/OR**<br>• Minor damage to tooth structure	• Able to achieve correct access outline with prompts<br>**AND**<br>• No damage to tooth structure	• Able to achieve correct access outline without prompts<br>**AND**<br>• No damage to tooth structure
Deroofing	• Failure to deroof pulp chamber entirely despite being prompted<br>**AND/OR**<br>• Major damage to pulpal walls or any damage to floor	• Able to deroof pulp chamber entirely with prompts<br>**AND/OR**<br>• Minor damage to pulpal walls	• Able to deroof pulp chamber entirely with prompts<br>**AND**<br>• No damage to pulpal walls or floor	• Able to deroof pulp chamber entirely without prompts<br>**AND**<br>• No damage to pulpal walls or floor

Supplementary Table S2: Student perceptions of pre-clinical haptic virtual reality simulation training

	Responses (%)				
**Statement**	Strongly agree	Agree	Neutral	Disagree	Strongly disagree
Pulpotomy demonstration on the HVRS device allowed me to clearly comprehend the tasks expected from me					
Images of the teeth, pulp chamber, and instruments displayed on the HVRS monitor looked realistic					
I could differentiate between the texture and hardness of enamel and dentine in the HVRS device					
Tactile force feedback given by the HVRS device felt realistic					
Deroofing the pulp chamber on the HVRS device felt similar to that on plastic teeth mounted on mannequins					
Training on the HVRS device improved my fine motor dental skills					
Training on the HVRS device improved my confidence in performing the pulpotomy procedure					
HVRS can replace conventional pre-clinical training on plastic teeth for the pulpotomy procedure					
HVRS can supplement conventional pre-clinical training on plastic teeth for the pulpotomy procedure					
I would like to have more HVRS sessions for paediatric pre-clinical pulp therapy procedures					

Open-ended questions

1. In your opinion what is the main benefit of HVRS training?
2. In your opinion what is the main limitation HVRS of training?
3. How could pre-clinical training for pulpotomy in primary teeth be improved in the future?
4. Would you prefer to have HVRS training for primary molar pulpotomy before or after training on plastic tooth models?
